# Analysis of Economic Activity Participation and Determining Factors Among Married Women by Income Level After the COVID-19 Pandemic

**DOI:** 10.3390/bs15040399

**Published:** 2025-03-21

**Authors:** Yu-Jin Cha

**Affiliations:** Department of Occupational Therapy, Semyung University, Jecheon 27136, Republic of Korea; occujin@semyung.ac.kr

**Keywords:** COVID-19 pandemic, economic activity participation, income level, Korean women, married women

## Abstract

This study examines the economic activities of married women aged 20 and above after the COVID-19 pandemic, focusing on variations across income levels. Using data from the 2022 Social Survey by Statistics Korea, which includes a nationally representative sample of over 38,000 individuals from 19,000 households, this study investigates the determinants of married women’s participation in economic activities and their impact on life satisfaction. Key variables, including employment status, income level, and life satisfaction, are measured using validated survey instruments. Hierarchical and multiple regression analyses are employed to assess how income levels moderate these effects, while correlation analysis is conducted to address multicollinearity concerns and ensure the robustness of the results. Findings indicate that income level has a significant but varying influence on the relationship between economic participation and life satisfaction. Lower-income women exhibit greater sensitivity to employment status in terms of life satisfaction, whereas higher-income women’s satisfaction is more influenced by social status and cultural factors. Additionally, education and household conditions emerged as critical determinants of economic engagement across different income groups. These results underscore the need for income-specific policy interventions to support married women’s workforce participation post-pandemic. However, as this study relies on cross-sectional survey data, causality cannot be definitively established. Policymakers should consider differentiated strategies that address financial constraints, work–family balance, and cultural expectations to foster greater economic inclusion.

## 1. Introduction

The COVID-19 pandemic has severely impacted social, economic, and individual lives around the world. Women, especially mothers, have been disproportionately affected by the pandemic (Remery et al., 2022). The COVID-19 pandemic has led to unprecedented declines in employment as key economic activities, including jobs and children’s schooling, have been curtailed in the name of public health. Importantly, women’s employment has fallen more than men’s in this recession (Lim & Zabek, 2023).

South Korea’s persistently low birth rate poses serious socio-economic challenges, closely linked to the employment stability of married women. Despite various supportive policies, fertility rates have not improved, underscoring the urgent need to enhance women’s economic participation as a practical solution (Seo & Kim, 2021). Key determinants include economic stability, employment security, social support, gender equality perceptions, and policy interventions (Yoon & Park, 2024). Employment stability facilitates women’s return to work after childbirth, whereas job insecurity increases the likelihood of career interruptions, leading many to delay or forgo having children (Gurín, 2023). Additionally, insufficient childcare infrastructure and workplace discrimination exacerbate low birth rates, while greater male participation in childcare positively influences women’s childbirth decisions (Choi et al., 2023). Addressing this issue requires policies that enhance economic opportunities for women, expand childcare support, and promote gender equality in both the workplace and household (Seo & Kim, 2021).

While women’s labor force participation has increased over the years, the expectation that they bear primary responsibility for childcare and household duties remains strong (Yoon & Park, 2024). This traditional influence, reinforced by societal norms and workplace cultures, often leads women to reduce working hours, take career breaks, or exit the workforce entirely after childbirth. Husbands’ attitudes toward women’s work vary, but many still prioritize their wives’ domestic roles over professional aspirations, particularly in conservative households. Key policies include maternity and parental leave programs, the expansion of public childcare services, the implementation of flexible work arrangements, and financial support for child-rearing. While these policies help reduce the burden of childcare and promote women’s workforce participation, their impact on increasing fertility rates is shaped by complex interactions with broader socio-economic and cultural factors, rather than by policy support alone (Seo & Kim, 2021).

This study underscores how married women’s workforce participation is shaped by factors such as income levels, household responsibilities, and societal norms, all of which influence life satisfaction and family dynamics. By analyzing the determinants of economic activity across different income groups, this research provides targeted policy recommendations to reduce gender disparities, enhance employment stability, and contribute to national strategies for addressing the ongoing decline in birth rates.

Married women face significant challenges in balancing their roles at work and home, with factors such as childcare responsibilities, household labor division, and co-residence with parents greatly influencing their economic activity (J. W. Lee, 2020). The COVID-19 pandemic further exacerbated these difficulties, leading many to lose jobs, temporarily retire, or reduce working hours. In response, some adapted by embracing flexible work models facilitated by online education, remote work, self-employment, and entrepreneurship, highlighting the multifaceted impact of the pandemic on their workforce participation. In South Korea, where declining birth rates closely intersect with women’s employment stability, understanding and addressing the determinants of married women’s economic participation is critical (Remery et al., 2022). This study explores these complexities and proposes policy recommendations to support their integration into the labor market.

The higher a husband’s income, the less time his wife spends in the labor force (Kwak & Choi, 2015). Married women in low-income households may participate in the labor market out of economic necessity, whereas those in middle-income and higher-income households may not be as active in the labor market, as they have more flexibility to adhere to traditional gender roles (Chang & Cheon, 2014). Over the past two decades, growth in South Korea’s economic activity participation rate has been driven by women, and this growth can be attributed to an increase in the participation rate of married women rather than single women (Hong, 2025).

Women who experienced job loss or change due to the COVID-19 pandemic found that they were more likely to experience a job change if they were contingent workers, and the COVID-19 pandemic brought about various changes in the labor market, daily life, and family relationships (Y. R. Oh, 2022). The COVID-19 pandemic has made it more difficult for married women to balance their work and family roles, which in turn can affect their economic activity, with differential impacts across income levels. Family factors such as the number of children and hours of household work, which have been identified as constraints on women’s economic activity participation, may increase the likelihood of women dropping out of the labor market.

The COVID-19 pandemic caused profound disruptions across society, the economy, and individual lives, with women—particularly married women—disproportionately affected. Widespread job losses, the expansion of remote work, and interruptions to children’s education significantly reshaped the economic activity patterns and daily lives of married women (Albanesi & Kim, 2021; Collins et al., 2021). This study examines the post-pandemic period to assess the impact of these changes on married women’s economic participation and to explore the long-term implications for women’s employment. The findings aim to provide valuable insights for policymakers to develop strategies addressing these challenges.

## 2. Literature Review

### 2.1. Life Satisfaction and Participation in Economic Activities of Married Women

Married women who are employed have been shown to have higher levels of life satisfaction than housewives, even though they may experience conflicts with their multiple roles as they juggle household chores and raising children (G. S. Kim & Yoon, 2009). Married women’s participation in economic activities not only increases the family’s income and thus revitalizes the household economy, but also provides them with the opportunity to play a positive role as members of society and increase their personal life satisfaction (Jin, 2003). Having a job improves quality of life (QoL) (Purba et al., 2021). Job insecurity negatively impacts physical (e.g., somatic symptoms, pain), psychological (e.g., anxiety, depression, affect), and social well-being (e.g., social support, marital discord) (Nella et al., 2015). A study conducted in the United Kingdom revealed that employment reduces the risk of psychiatric disorders and loneliness (Li & Wang, 2020). Women’s employment also positively affects life satisfaction because it puts them on a more equal footing with their husbands in the marriage, which promotes gender equality and changes the balance of power within the family (S. H. Park & Lee, 2021).

### 2.2. Women’s Economic Activity Participation After the COVID-19 Pandemic

COVID-19 has significantly impacted everyone’s lives, leading to changes in how we live and work. The global economy has been affected, and a new normal has been established. In terms of the labor market impact, this research indicates that parents who have lost a job or experienced reduced working hours are more concerned about their children’s mental health than those whose employment status remained stable. (Abdo Ahmad et al., 2023). The heterogeneous findings between mothers and fathers are in agreement with earlier works on the gender pay gap and the double burden that women must balance between work and household activities, despite the literature showing a positive effect of women workers on firm performance (Terjesen et al., 2016). Given the relatively slow progress in reducing the gender employment gap, major international organizations have predicted that the COVID-19 pandemic will exacerbate discriminatory conditions for women’s economic activities, and have identified addressing gender inequality in disaster situations as a challenge (Erinn et al., 2021).

The COVID-19 pandemic has centralized traditional domestic roles for women, disrupted their professional work rhythm through teleworking, and increased rather than reduced the burden with husbands staying at home (Çoban, 2022). The dual reinforcement of traditional gender roles poses a risk of alienating women from employment, careers, and professional identities (Emslie & Hunt, 2009). During past epidemics, including SARS, swine flu, and bird flu, the negative impacts persisted even after men’s incomes rebounded. Without proactive interventions addressing gender disparities in pandemic responses, some women may never fully recover their lifetime earnings (Power, 2020).

### 2.3. Characteristics of Women’s Participation in Economic Activity in South Korea

In consistent findings, Statistics Korea’s Economically Active Population Survey showed that the female employment rate decreased by 1.6 percentage points after the COVID-19 pandemic, while the male employment rate decreased by 1.3 percentage points (Nam & Lee, 2020). Since South Korea has a poorer gender structure than other countries, the gender gap in this pandemic crisis may be more pronounced here than in other countries (Ham, 2021). Women who experienced job loss or change due to the COVID-19 pandemic found that they were more likely to experience job change if they were contingent workers, and the COVID-19 pandemic brought about various changes in the labor market, daily life, and family relationships (Y. R. Oh, 2022).

### 2.4. Factors Influencing the Participation in Economic Activity of Married Women

Previous research on the determinants of economic activity among married women has shown that a stable and supportive family structure increases the likelihood that married women will participate in economic activity (Y. R. Oh, 2022), and that married women with higher levels of education are more likely to participate in economic activity (Jin et al., 2011). It has also been reported that married women in economically stable situations are more likely to participate in economic activities (S. J. Kim & Kim, 2007). Women’s economic activity participation plays an important role in the balance and development of society, especially for married women, and it is therefore necessary to better understand its significance. However, there has been a lack of research on the status of married women’s economic activity and its determinants since the COVID-19 pandemic.

Married women are at the center of the debate on women’s economic activity participation in South Korea (Kwak & Choi, 2015). Women’s participation in economic activities is important to the development and balance of society, and the economic activities of married women is becoming a more seriously studied topic. Therefore, the purpose of this study is to investigate the economic activity status of married women and its determinants by income levels after the COVID-19 pandemic, and to suggest directions for social support and policy interventions. In particular, this study is expected to provide new insights into women’s economic situation after the COVID-19 pandemic and contribute to making informed policy decisions. In addition, this study will make an important contribution to raising awareness of social inequality and exploring policy interventions to resolve it by analyzing the differential impact on married women’s economic activities by income levels. In doing so, this study is anticipated to provide new perspectives and information to academics and policymakers.

## 3. Research Framework and Hypotheses Development

**Figure 1:**
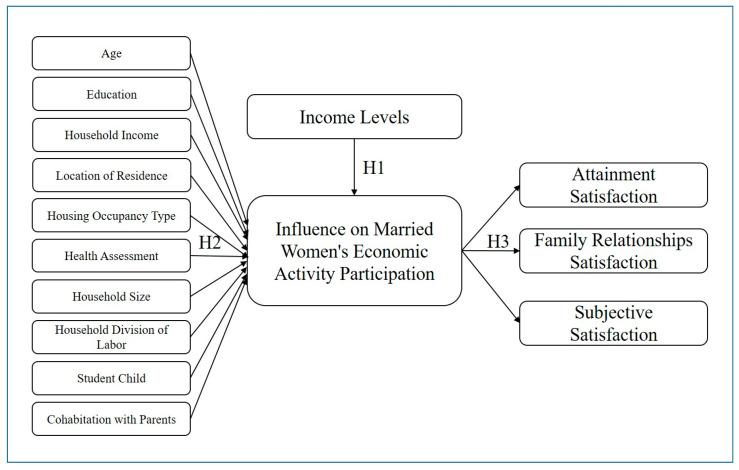
Research model.

Building on the existing literature on the economic activities of married women, this study recognizes a correlation between their employment status and life satisfaction, which is influenced by income levels. To explore these relationships empirically, this study examines how married women’s participation in economic activities varies across different income levels, how the factors influencing their participation differ by income levels, and how their life satisfaction is shaped by both economic activity participation and income levels. The research model, based on the above theoretical framework, is presented in Figure 1.

## 4. Method and Data Resource

### 4.1. Collecting Data

This study is a secondary analysis of the raw data of the 2022 Social Survey provided by the Microdata Integrated Service (MDIS) of Statistics Korea (2023). The Social Survey is a survey conducted by the National Statistical Office and covers five sectors (welfare, social participation, culture and leisure, income and consumption, and labor) and five categories (health, education, safety, family, and environment) on an annual rotating basis (J. O. Kim & Park, 2021). The Social Survey was conducted by interviewing more than 38,000 people in 19,000 sample households using the probability proportional to size samplings (PPS) method in 17 provincial administrative districts based on census tracts and 27 regions stratified by towns and villages (Dong-eup-myeon) (Min, 2011).

### 4.2. Setting Variables

#### 4.2.1. Demographic Characteristics

The demographic characteristics and control variables selected for this study were based on previous research (Kwak & Choi, 2015). The variables included age, education, household income, location of residence, housing occupancy type, health assessment, household size, household division of labor, presence of a student child, and cohabitation with parents. Age was categorized into under 20, 19–29, 30–39, 40–49, 50–59, and 60+ years. Education level was classified as unschooled, elementary school, middle school, high school, college (less than 4 years), university (4+ years), master’s program, and doctoral program. Household income was divided into less than 1 million won, 1–2 million won, 2–3 million won, 3–4 million won, 4–5 million won, 5–6 million won, 6–7 million won, 7–8 million won, and more than 8 million won. Location of residence was recorded as a categorical variable. For employment-related factors, wage earners were categorized as business employees, interim workers, and daily laborers. Economic activity status was classified into employment, unemployment, and economic inactivity. Work-from-home status related to COVID-19 was categorized as yes or no. Regarding housing and household characteristics, housing occupancy type was categorized as own, key money, monthly rent with deposit, monthly rent without deposit, and free of charge. Health assessments were classified as very good, good, fair, poor, and very poor. Household size, household division of labor, presence of a student child, and cohabitation with parents were also considered as key control variables in the study.

#### 4.2.2. Social Environment Factors

Variables used to investigate the social environment factors of the respondents included household division of labor, presence of student children, and cohabitation with parents. Household division of labor was categorized into five categories: wife takes full responsibility, wife primarily handles and husband contributes, shared responsibilities, husband primarily handles and wife contributes, and husband takes full responsibility. The presence of student children was selected as yes or no. Cohabitation with parents was categorized into two categories: living together and not living together.

#### 4.2.3. Income Bracket Classifications

Household equalized income is a value adjusted for the number of household members to compare income levels across households, and is calculated as household income divided by the square root of the number of household members (Nam & Lee, 2020). The calculated household equalized income is divided into low, middle, and upper income groups based on 50% and 150% of the median income. Previous studies, including the OECD, have categorized income groups as low income who are below 50% of median income, middle income for those between 50–150%, and upper income for those above 150% (Atkinson, 2007; S. H. Park & Lee, 2021). The middle income in 2022 is based on KRW 3,269,085 for 2 people, KRW 4,194,701 for 3 people, and KRW 5,121,080 for 4 or more people, based on data from Statistics (Statistics Korea, 2023).

### 4.3. Analytic Strategies

This study conducts a frequency analysis of the demographic and social environment factors of the study subjects. In this study, hierarchical regression and multiple regression models were employed for regression analysis. A hierarchical regression analysis was conducted to explore the variations in satisfaction levels among married women according to their employment status and income class, while multiple regression analysis was employed to identify the factors influencing married women’s engagement in economic activities across three income groups: low-income, middle-income, and upper-income. Specifically, the independent variables in the regression analysis were demographic characteristics and social environment factors, and the dependent variables were economic activity status (employment, unemployment, and inactivity). Correlation analysis was conducted to test the correlation between the main variables and to check for the problem of multicollinearity. By checking the correlations and multicollinearity problems of key variables, the reliability and interpretability of the regression analysis results are improved.

The study addresses potential endogeneity issues through a robust methodological framework. Hierarchical and multiple regression analyses were employed to explore variations in satisfaction levels and identify the determinants of married women’s economic activity participation across income groups. Additionally, correlation analysis was conducted to test for multicollinearity among key variables, ensuring the reliability and interpretability of the results. By categorizing variables such as household income, regional classification, and social environment factors, the study controls for confounding influences that could bias the findings. These steps, combined with the use of large-scale, nationally representative data from the 2022 Social Survey, contribute to minimizing endogeneity concerns and enhancing the validity of the conclusions. Statistical analyses were conducted using SPSS version 28, with statistical significance set at *p* < 0.05 for all tests.

## 5. Results

### 5.1. Descriptive Results

The demographic characteristics of the post-COVID-19 married women data collected in this study by income group are as follows. In the low-income segment, the majority of participants were aged 60 or older, with a significant portion having elementary school education and a household income below 1 million won. Most resided in the Dong region and were economically inactive, with some working from home due to COVID-19. The household size was typically two persons, with the wife primarily managing household duties. A high percentage were childless or students and did not cohabit with their parents.

Among the middle-income segment, participants were generally in their 50s or older, with a considerable number having graduated from high school and earning between 2 and 3 million won. Similar to the low-income group, many lived in the Dong region and were economically inactive, with some working remotely due to the pandemic. Household sizes were typically around two persons, with wives predominantly handling household responsibilities. A significant proportion of respondents, particularly in the upper-income group, were in their 50s, had completed high school, and earned over 8 million won. Most resided in the Dong region and were employed, and some worked from home due to COVID-19. A large percentage were childless or students who did not cohabit with their parents (Table 1).

### 5.2. Regression Results

This study examines variations in satisfaction levels among married women based on income groups and employment statuses. The findings can be summarized as follows: Among women in the low-income group, those who were employed reported higher levels of satisfaction in terms of family relationships compared to those who were not employed. However, there were no significant differences between the two groups in terms of attainment satisfaction and subjective satisfaction. In the middle-class category, there were no significant differences in attainment satisfaction, family relationship satisfaction, and subjective satisfaction based on employment status. Within the upper-income group, employed women exhibited higher levels of satisfaction in terms of family relationships compared to their non-employed counterparts. Similar to the previous groups, there were no significant differences in attainment satisfaction and subjective satisfaction. These findings highlight the influence of income group and employment status on satisfaction levels, particularly in the context of family relationships (Table 2).

This study employed logistic regression analysis to investigate the determinants impacting the involvement of married women in economic activity. Findings from the logistic regression analysis within the low-income bracket revealed that household income, location of residence, and the presence of school-age children were influential factors affecting the economic engagement of married women. Specifically, higher household income was associated with increased participation in economic activities, greater participation was observed in urban areas compared to rural areas, and married women with school-age children exhibited higher participation in economic activities compared to those without children.

Subsequently, an analysis was conducted on the middle-income group, which revealed that factors such as age, household income, location of residence, health assessment, household size, household division of labor, presence of a student child, and cohabitation with parents were influential in determining the economic activity participation of married women. Specifically, higher age and household income were associated with increased economic activity participation. Additionally, individuals residing in towns and villages were more likely to engage in economic activities compared to those in cities, and those with better health assessments also exhibited higher participation. Moreover, women in households with fewer members and where husbands contributed more to household chores were more likely to participate in economic activities. Furthermore, married women with school-age children and those living with their parents were also more likely to be economically active.

Finally, when analyzing the upper-income group, we found that age, household income, location of residence, and household division of labor influenced economic activity participation among married women: younger age was associated with higher economic activity participation, and higher household income was associated with higher economic activity participation. In terms of region, towns and villages were more likely to participate in economic activities than cities, and husbands were more likely to participate in economic activities the more they shared household chores (Table 3).

A correlation analysis was performed to examine the relationships between the variables in the study. The findings revealed that the majority of the variables were significantly correlated with one another. Life satisfaction (including attainment satisfaction, family relationship satisfaction, and subjective satisfaction) was found to have significant positive correlations with age, housing occupancy type, and health assessment (*p* < 0.01). However, it showed significant negative correlations with education, household income, household size, household division of labor, and having a student child (*p* < 0.01). In other words, older age, living in one’s own home, having good health, lower education level, lower household income, smaller household size, less household division of labor, and having a student child are all factors that contribute to higher life satisfaction. Furthermore, all correlation coefficients were below 0.8, indicating the absence of multicollinearity (Table 4).

## 6. Discussion

Over the past two decades, the increase in women’s economic activity in South Korea has been primarily driven by married women rather than single women, highlighting their increasing role in the labor market (H. J. Park & Lee, 2004). Traditional gender roles and family responsibilities significantly influence married women’s employment decisions, especially in lower-income groups (Kang et al., 2024). However, this study identifies income levels as a key determinant of labor market participation, shifting the focus beyond the cultural constraints emphasized in previous research.

Previous research has consistently demonstrated that employment status influences family relationship satisfaction among married women, with variations across income groups due to economic constraints and social structures (Bae & Kim, 2012; Blau & Kahn, 2007; Evlilik & Doyumu, 2017; J. Y. Kim & Shin, 2022). For instance, Cha (2022) found that in lower-income households, where financial stress is high, women’s employment contributes significantly to household stability, thereby improving family relationship satisfaction (Cha, 2022). Similarly, Yoon and Park (2024) highlighted that middle-income women often experience greater work–family conflict, making their employment less likely to enhance overall satisfaction (Yoon & Park, 2024).

In contrast to these findings, this study reveals that employment status significantly increases family relationship satisfaction only in low- and high-income groups, whereas middle-income women show no substantial differences based on employment. This suggests that for middle-income women, employment may not necessarily enhance well-being, due to competing pressures between financial stability and household responsibilities. While previous studies emphasized childcare availability as a key determinant of women’s employment decisions (Yoon & Park, 2024), the results of this study indicate that income level itself serves as a stronger moderating factor, particularly for middle-income women, whose employment-related satisfaction does not significantly differ from that of their non-employed counterparts.

These findings contribute to the broader discussion on the interaction between economic status and employment satisfaction. Unlike prior research that predominantly focused on external support systems, such as government childcare subsidies and workplace flexibility policies (Seo & Kim, 2021), this study highlights that women’s employment satisfaction is highly dependent on intrinsic economic stability rather than institutional factors alone. Therefore, income-specific policy interventions should be designed to address the distinct economic pressures experienced by different income groups, rather than applying a uniform approach to employment support programs.

In the low-income group, the economic burden at home is likely to be higher, so the employment status of married women is expected to be closely related to the economic stability of the family. The impact of married women’s employment on family relationship satisfaction is expected to be greater in low-income households. However, in upper-income households, economic stability is likely to be relatively high, resulting in less financial burden at home (Cha, 2022).

Therefore, married women’s employment status may be less directly related to household economic stability, which is expected to have a smaller effect on family relationship satisfaction. The middle-income contingent is an economically stable and self-respecting group, for whom employment status may not have a significant impact on achievement or family relationships due to the relatively high economic stability. Therefore, the individual circumstances and values of each family should be considered to determine the impact on family relationships.

Second, logistic regression analysis was conducted for each income group. In the low-income group, household income, regional division, and the presence of student children influenced married women’s participation in economic activities. In the middle-income group, additional factors such as age, health assessment, household size, household division of labor, student status, and parental cohabitation also played a role. Finally, in the upper-income group, age, household income, regional division, and household division of labor were significant predictors. Given these findings, it is essential to assess the model’s overall explanatory power. The following discussion examines the adequacy of the logistic regression models by evaluating their Nagelkerke R^2^ values and statistical significance.

Although the Nagelkerke R^2^ values in this study appear relatively low, this is common in logistic regression models (Menard, 2000). Unlike OLS regression, pseudo-R^2^ values in logistic regression do not directly measure variance explained but rather indicate improvements over the null model. Despite their modest size, the statistically significant χ^2^ values confirm that the predictors meaningfully contribute to explaining married women’s employment participation. Additionally, the Nagelkerke R^2^ values vary across income groups, with a relatively higher value observed in the upper-income group (R^2^ = 0.342), suggesting a stronger model fit in this segment. While the models provide valuable insights, they may benefit from additional explanatory variables to capture the complexities of married women’s labor market participation more comprehensively.

Future research should explore longitudinal data to better capture the dynamic impact of income levels on married women’s labor market participation. Additionally, qualitative studies could provide deeper insights into how socio-cultural expectations influence employment decisions across different income groups.

An analysis of the relationship between income and labor market participation among married women found that the higher the income of married women, the more likely they are to participate in the labor market. In particular, families with higher incomes tend to have greater economic stability, which leads to higher rates of labor market participation among women (Sudo, 2017). On the other hand, a higher monthly income of the spouse is generally found to reduce the probability of reemployment for women (K. N. Park, 2009). This can be interpreted to indicate a division of labor between men and women when the spouse’s monthly income is high, thus maintaining the male-centered single breadwinner model.

Married women in higher income brackets usually have a stable economic situation, and the presence or absence of children may not have a significant impact on their employment (Goldin et al., 2022). This is because it is possible to pay for childcare and maintain employment at the same time, and because upper-income women often have stable jobs, such as professional jobs, that allow them to outsource housework and childcare, or to be grandparents (Ye, 2013). On the other hand, for married women in the middle-income group, the presence or absence of children may influence their employment status. Middle-income women may be torn between the cost of caring for their children and maintaining employment, which may lead them to forgo employment (Yoon & Park, 2024).

Married women in low-income households can face many financial pressures, and the presence or absence of children can have a significant impact on their ability to work (Chang & Cheon, 2014). Among low-income families, mothers are more likely to be employed when they have access to childcare options (Ruppanner et al., 2019), and not only are they more likely to sacrifice employment when they have childcare problems (Maume, 2008), but women living with children experience larger declines in economic activity participation and are more likely to leave the labor force. For mothers with school-age children, these effects are concentrated among low-income workers (Lim & Zabek, 2023). It can be very difficult to pay for the care of children and maintain employment at the same time, and many women may choose not to work.

For this reason, the extent to which the presence of children affects the employment status of married women may vary across income groups, and it is important that policies and support systems take this into account (Blau & Kahn, 2007). An important aspect of understanding married women’s economic participation is considering the presence of children as a determining factor (Gurín, 2023). As highlighted in this study, the impact of having children on employment status varies across income groups. In low-income households, mothers are more likely to work if they have access to childcare services, as economic necessity drives their workforce participation (Seo & Kim, 2021). On the other hand, middle-income women may experience more conflict between childcare costs and maintaining employment, sometimes leading them to exit the workforce (Yoon & Park, 2024). For higher-income women, the presence of children tends to have a less significant effect on employment, due to greater financial flexibility and access to outsourced childcare options. Given these variations, future studies should explore more refined datasets that distinguish between the presence and age of children, allowing for a deeper analysis of their role in labor market participation. Moreover, policymakers should design targeted support systems that acknowledge these differences, ensuring that childcare and economic assistance are tailored to the needs of each income group.

Current support policies in South Korea include various programs aimed at facilitating married women’s participation in economic activities, regardless of whether they have children (G. S. Kim & Yoon, 2009). However, there remains a need for further discussion on whether these policies adequately account for differences across income levels. Key policies include maternity and parental leave, the expansion of public childcare services, the implementation of flexible work arrangements, and financial support for child-rearing. However, as demonstrated by the findings of this study, these policies do not have the same effect across all income groups. For instance, low-income women often enter the labor market out of economic necessity, and access to public childcare services can be a critical factor in determining their employment status (Sandstrom & Chaudry, 2012).

In contrast, for middle-income women, household financial conditions may play a greater role in labor market participation than childcare availability (Yoon & Park, 2024). Therefore, future policy design should incorporate differentiated support based on income levels, with an emphasis on expanding childcare and financial assistance for low-income families, as these measures are likely to play a crucial role in promoting married women’s workforce participation. Studies examining whether labor market outcomes are negatively affected by parents and young children find that while the number of children per se does not determine labor market outcomes, the duration of interaction between children and women explains the transition to part-time work, reduced paid working hours, and unemployment (Cowan, 2020; Montenovo et al., 2022).

There is mixed evidence that older married women have higher economic activity participation rates than younger married women (H. S. Park & Ra, 2009) and that economic activity participation rates increase up to a certain age and then decline (Choi & Yoo, 2009). A study on the relationship between age and economic activity participation among married women found that the older the woman, the less likely she is to be employed as a wage earner and the more likely she is to be self-employed (E. J. Oh et al., 2009). Age is a significant determinant of women’s economic activity participation, but the association cannot be explained by a linear correlation (Jin et al., 2011).

In terms of location of residence, the results are consistent with existing studies that show that those who live in large cities have higher rates of economic activity participation (Ju, 2010). This is due to the fact that urban areas offer diverse industries and corporations, presenting a broad spectrum of job prospects. Additionally, these areas provide an array of childcare facilities and centers, alleviating the childcare responsibilities of married women. This reduces the burden of caring for children when looking for employment, allowing mothers to participate in economic activities. Due to these factors, the economic activity participation of married women residing in urban areas may be linked to increased economic activity, and studies indicate that a range of environmental and social factors interact to impact this.

A study by the Korea Labor Institute (KLI) found that married women in Korea are not only limited in their employment opportunities due to the burden of domestic work, but also find it difficult to maintain both domestic work and employment at the same time (Bae & Kim, 2012). In the existing research on the impact of household workload on the employment of married women, most studies show that household workload has a negative impact on women’s employment. In particular, it has been found that women in high-duty households experience more stress at work, have lower job satisfaction, and may be constrained in their career advancement (J. Y. Kim & Shin, 2022). In addition, studies have shown that women in high-duty households may find it difficult to balance work and family, which in turn may reduce their performance and achievement at work (S. E. Oh & Kim, 2020). The findings indicate that the employment status of married women in South Korea is influenced by the division of labor. This suggests that the burden of domestic work acts as a constraint on employment opportunities for married women. Therefore, there is a need to implement policies and support systems aimed at reducing the burden of domestic work.

This study utilizes secondary data, which presents certain limitations. First, the reliance on secondary data limited the scope of examining the effects of various policy measures on women’s economic activities, such as the expansion of parental leave, the introduction of flexible work systems, and government subsidies. Second, as this study was conducted in 2022, a period when the COVID-19 pandemic had a profound impact on women’s employment, it does not allow for a direct comparison with pre-pandemic labor market conditions.

The primary objective of this study is to analyze post-pandemic labor market conditions and identify key determinants shaping women’s employment based on income levels. While a longitudinal analysis comparing pre- and post-pandemic trends would provide deeper insights, this study focuses on understanding how socio-economic factors have influenced the labor market participation of married women in the aftermath of COVID-19. To address this limitation, future research should incorporate pre-pandemic data to offer a more comprehensive perspective on long-term employment patterns among married women.

South Korea is facing a severe ultra-low-fertility crisis, raising concerns about the effectiveness of existing fertility rebound policies (Choi & Won, 2020). Despite various supportive measures, birth rates have remained stagnant, highlighting the need for a fundamental reassessment of policy efforts. One crucial aspect to consider is married women’s participation in economic activities, as it significantly impacts marriage rates, fertility, child-rearing, and elderly care (Liao, 2011). Research has shown that employment stability positively influences fertility decisions, emphasizing the importance of policy, business, and societal efforts to support women’s workforce participation (C. J. Lee, 2021). Rather than focusing solely on pro-natalist incentives, addressing structural barriers to women’s employment may offer a more sustainable approach to balancing economic activity and demographic stability.

The findings of this study reinforce the need for targeted policy interventions to enhance married women’s economic participation, particularly in the post-pandemic era. Given that economic constraints, employment stability, and household responsibilities shape women’s workforce participation, policymakers should implement income-specific strategies. For lower-income women, financial assistance, job training, and affordable childcare services can alleviate economic pressures and facilitate stable employment opportunities (Seo & Kim, 2021). Middle-income women may benefit from work–life balance initiatives, such as flexible working hours, improved parental leave policies, and incentives for employers to cultivate family-friendly workplace environments (Yoon & Park, 2024). Meanwhile, higher-income women require policies that dismantle structural barriers related to gender biases in career advancement, ensuring that highly skilled women can fully engage in the workforce without being disproportionately burdened by traditional family expectations (Gurín, 2023). Additionally, fostering gender-equitable labor policies, including paternity leave incentives and workplace cultural shifts, can enhance long-term workforce participation and reduce gender disparities (Choi et al., 2023). Addressing these challenges demands a comprehensive approach that integrates financial, social, and cultural factors to create a more inclusive labor market for married women.

## 7. Conclusions

This study analyzed the status and determinants of married women’s economic activity across income groups in the post-COVID-19 period. The results indicate that in both low-income and upper-income groups, employed women reported higher family relationship satisfaction than non-employed women, while there were no significant differences in attainment satisfaction and subjective satisfaction. In contrast, among middle-income women, employment status was not associated with differences in satisfaction levels. Furthermore, the factors influencing married women’s participation in economic activities varied significantly by income level, underscoring the need for differentiated policy approaches.

Despite its contributions, this study is limited by its reliance on secondary data, which restricts the depth of interpretation. Future research should incorporate a broader range of variables and a larger sample size to provide a more comprehensive analysis. Additionally, qualitative studies could offer deeper insights into the socio-cultural factors shaping married women’s labor market participation. These findings highlight the importance of policies that enhance married women’s economic participation as a strategy to address Korea’s ultra-low birth rate. Given that the determinants of labor market participation differ across income groups, policymakers should develop targeted interventions that account for these variations. Ensuring equitable employment opportunities and tailored support systems will be crucial in fostering an inclusive labor market for married women.

This study contributes to the understanding of the challenges faced by married women during the post-pandemic economic disruption and offers policy recommendations to mitigate these challenges. Unlike previous studies that focused on overall employment trends, this research emphasizes income levels as a key moderating factor in work–family dynamics, providing a nuanced perspective on the structural barriers affecting women’s economic participation in South Korea.

## Figures and Tables

**Table 1:** Demographic characteristics.

	Variable	Items	Low Income		Middle Income		Upper Income		Total	
			N	%	N	%	N	%	N	%
Total			1078	100.0	5420	100.0	3976	100.0	10,474	100.0
Personal factors	Age	Mean (SD)	65.42	(13.078)	54.16	(13.684)	50.23	(11.114)	53.83	(13.422)
		Under age 20	0	0	0	0	1	0	1	0
		20–29	9	0.8	95	1.8	104	2.6	208	2
		30–39	53	4.9	810	14.9	646	16.2	1509	14.4
		40–49	72	6.7	1306	24.1	1085	27.3	2463	23.5
		50–59	146	13.5	1062	19.6	1305	32.8	2513	24
		Age 60 or older	798	74.0	2147	39.6	835	21.0	3780	36.1
	Education	Unschooled	108	10	100	1.8	7	0.2	215	2.1
		Elementary school	382	35.4	725	13.4	149	3.7	1256	12
		Middle school	205	19	744	13.7	225	5.7	1174	11.2
		High school	251	23.3	1900	35.1	1345	33.8	3496	33.4
		College (less than 4 years)	65	6	932	17.2	725	18.2	1722	16.4
		University (4+ years)	56	5.2	894	16.5	1189	29.9	2139	20.4
		Master’s program	11	1	109	2	279	7	399	3.8
		Doctoral program	0	0	16	0.3	57	1.4	73	0.7
	Household income	Less than 1 million won	779	72.3	0	0	0	0	779	7.4
		Less than 2 million won	299	27.7	1216	22.4	0	0	1515	14.5
		Less than 3 million won	0	0	1685	31.1	0	0	1685	16.1
		Less than 4 million won	0	0	1061	19.6	701	17.6	1762	16.8
		Less than 5 million won	0	0	974	18	465	11.7	1439	13.7
		Less than 6 million won	0	0	484	8.9	684	17.2	1168	11.2
		Less than 7 million won	0	0	0	0	653	16.4	653	6.2
		Less than 8 million won	0	0	0	0	457	11.5	457	4.4
		8 million won and above	0	0	0	0	1016	25.6	1016	9.7
	Location of residence	Dong	691	64.1	3885	71.7	3068	77.2	7644	73
		Eup-myeon	387	35.9	1535	28.3	908	22.8	2830	27
	Wage earners	Business employees	63	5.8	1059	19.5	1497	37.7	2619	25
		Interim workers	59	5.5	453	8.4	258	6.5	770	7.4
		Daily laborers	25	2.3	121	2.2	71	1.8	217	2.1
	Economic activity	Employment	280	26	2569	47.4	2485	62.5	5334	50.9
		Unemployment and economic inactivity	798	74	2851	52.6	1491	37.5	5140	49.1
	Work from home, whether COVID-19-related	Yes	22	2	253	4.7	482	12.1	757	7.2
		No	5	0.5	66	1.2	86	2.2	157	1.5
	Housing occupancy type	Own	801	74.3	4125	76.1	3085	77.6	8011	76.5
		Key money	101	9.4	613	11.3	517	13	1231	11.8
		Monthly rent with deposit	127	11.8	534	9.9	290	7.3	951	9.1
		Monthly rent without deposit	11	1	26	0.5	15	0.4	52	0.5
		Free of charge	38	3.5	122	2.3	69	1.7	229	2.2
	Health assessment	Very good	36	3.3	316	5.8	331	8.3	683	6.5
		Good	232	21.5	2054	37.9	1906	47.9	4192	40
		Fair	444	41.2	2323	42.9	1485	37.3	4252	40.6
		Poor	308	28.6	678	12.5	238	6	1224	11.7
		Very poor	58	5.4	49	0.9	16	0.4	123	1.2
	Household size	1 person	0	0	0	0	0	0	0	0
		2 persons	679	63	2156	39.8	1985	49.9	4820	46
		3 persons	273	25.3	1383	25.5	1025	25.8	2681	25.6
		4 or more	126	11.7	1881	34.7	966	24.3	2973	28.4
	Dual income	Dual-income	6	0.6	180	3.3	270	6.8	456	4.4
		Single-income	57	5.3	250	4.6	171	4.3	478	4.6
Social and environmental factors	Household division of labor	Wife takes full responsibility	344	31.9	1367	25.2	870	21.9	2581	24.6
		Wife primarily handles, husband contributes	476	44.2	2929	54	2118	53.3	5523	52.7
		Shared responsibilities	195	18.1	952	17.6	867	21.8	2014	19.2
		Husband primarily handles, wife contributes	50	4.6	151	2.8	99	2.5	300	2.9
		Husband takes full responsibility	13	1.2	20	0.4	21	0.5	54	0.5
	Student child	Yes	122	11.3	1906	35.2	1347	33.9	3375	32.2
		No	956	88.7	3514	64.8	2629	66.1	7099	67.8
	Cohabitation with parents	Living together	12	1.1	82	1.5	57	1.4	151	1.4
		Not living together	261	24.2	2853	52.6	2745	69	5859	55.9

Note: N, sample size. SD, standard deviation.

**Table 2:** Differences in satisfaction among married women by income group and employment status.

Category		Low Income		Middle Income		Upper Income	
		M	SD	M	SD	M	SD
Attainment satisfaction	Not employed	2.97	0.812	2.78	0.836	2.48	0.862
	Employed	3.05	0.903	2.79	0.848	2.52	0.870
	t-value	−1.287		−0.249		−1.278	
Family relationship satisfaction	Not employed	2.27	0.820	2.26	0.803	2.10	0.786
	Employed	2.41	0.767	2.23	0.807	2.17	0.813
	t-value	−2.505 *		1.274		−2.539 **	
Subjective satisfaction	Not employed	2.90	0.785	2.68	0.885	2.34	0.865
	Employed	2.96	0.915	2.64	0.874	2.33	0.902
	t-value	−1.076		1.558		0.359	

Note: M, mean. SD, standard deviation. * *p* < 0.05, ** *p* < 0.01.

**Table 3:** Factors influencing the economic activity participation of married women across income brackets.

	Low Income				Middle Income				Upper Income			
	B	S.E	Wald	Exp (β)	B	S.E	Wald	Exp (β)	B	S.E	Wald	Exp (β)
Age	0.019	0.017	1.214	1.019	0.029	0.005	38.503 ***	1.030	−0.013	0.005	7.224 **	0.987
Education	−0.141	0.132	1.134	0.869	−0.007	0.040	0.031	0.993	−0.036	0.040	0.817	0.965
Household income	0.871	0.369	5.583 *	2.389	0.236	0.043	30.422 ***	1.266	0.167	0.030	30.176 ***	1.182
Location of residence	−1.016	0.300	11.463 ***	0.362	−0.531	0.092	33.258 ***	0.588	−0.455	0.107	18.114 ***	0.634
Housing occupancy type	−0.049	0.130	0.145	0.952	−0.003	0.042	0.006	0.997	0.002	0.052	0.001	1.002
Health assessment	0.004	0.174	0.001	1.004	0.107	0.052	4.188 *	0.899	−0.031	0.057	0.297	0.969
Household size	0.118	0.261	0.204	1.125	−0.202	0.073	7.581 **	0.817	−0.125	0.070	3.144	0.883
Household division of labor	0.120	0.173	0.478	1.127	0.355	0.054	43.292 ***	1.426	0.521	0.059	77.489 ***	1.683
Student child	0.783	0.343	5.207 *	2.188	0.359	0.094	14.756 ***	1.433	0.052	0.101	0.259	1.053
Cohabitation with parents	0.505	0.627	0.648	1.657	0.580	0.243	5.697 *	1.786	0.172	0.299	0.330	1.187
	Number of cases = 1078 χ^2^ = 35.234 Nagelkerke R^2^ = 0.166				Number of cases = 5420 χ^2^ = 162.218 Nagelkerke R^2^ = 0.072				Number of cases = 1871 χ^2^ = 551.21 Nagelkerke R^2^ = 0.342			

Note: B, unstandardized regression coefficient. S.E, standard error. Wald, Wald statistic. Exp, exponentiated coefficient. β, standardized regression coefficient. * *p* < 0.05, ** *p* < 0.01, *** *p* < 0.001.

**Table 4:** Analysis of correlations among the primary variables.

Variable	Age	Education	Household Income	Location of Residence	Housing Occupancy Type	Health Assessment	Household Size	Household Division of Labor	Student Child	Cohabitation with Parents	Attainment Satisfaction	Family Relationship Satisfaction	Subjective Satisfaction
Age	1												
Education	−0.637 **	1											
Household income	−0.436 **	0.510 **	1										
Location of residence	−0.143 **	0.233 **	0.158 **	1									
Housing occupancy type	−0.150 **	0.066 **	−0.049 **	0.045 **	1								
Health assessment	0.339 **	−0.331 **	−0.254 **	−0.031 **	−0.009	1							
Household size	−0.469 **	0.343 **	0.394 **	0.135 **	0.021 *	−0.152 **	1						
Household division of labor	−0.141 **	0.117 **	0.040 **	0.037 **	0.056 **	−0.041 **	−0.006	1					
Student child	−0.448 **	0.372 **	0.331 **	0.103 **	0.026 **	−0.157 **	0.607 **	−0.022 *	1				
Cohabitation with parents	0.037 **	−0.005	0.011	−0.002	0.001	−0.012	0.089 **	−0.003	−0.020	1			
Attainment satisfaction	0.067 **	−0.162 **	−0.217 **	0.005	0.094 **	0.338 **	−0.023 *	−0.053 **	−0.037 **	0.006	1		
Family Relationship satisfaction	0.160 **	−0.145 **	−0.120 **	0.011	−0.007	0.227 **	−0.051 **	−0.127 **	−0.051 **	0.006	0.288 **	1	
Subjective satisfaction	0.130 **	−0.222 **	−0.247 **	−0.014	0.074 **	0.390 **	−0.055 **	−0.055 **	−0.074 **	−0.001	0.650 **	0.329 **	1

Note: * *p* < 0.05, ** *p* < 0.01

## Data Availability

The data presented in this study are available on request from the corresponding author.
